# Ethnobotanical survey of medicinal plant species used by communities around Mabira and Mpanga Central Forest Reserves, Uganda

**DOI:** 10.1186/s41182-021-00341-z

**Published:** 2021-06-29

**Authors:** Savina Asiimwe, Jane Namukobe, Robert Byamukama, Betty Imalingat

**Affiliations:** 1 Department of Plant Sciences, Microbiology & Biotechnology, Makerere University, P.O Box 7062, Kampala, Uganda; 2 Department of Chemistry, Makerere University, P.O Box 7062, Kampala, Uganda

**Keywords:** Ethnobotany, Medicinal plants, Conservation, Percent use-value informant consensus factor, Mabira, Mpanga, Uganda

## Abstract

**Background:**

Medicinal plants form an integral part of many health care systems in Uganda. This study aimed at documenting the therapeutic importance of plant species used in primary health care among communities living adjacent to Mabira and Mpanga forest reserves in Central Uganda.

**Methods:**

An ethnobotanical study was conducted between April and June 2018 in 7 villages adjacent to Mpanga and 6 villages adjacent to Mabira central forest reserves. Information was obtained from 28 respondents identified using snowball and purposive sampling techniques and interviewed using semi-structured questionnaires. Descriptive statistics were used to present the data. The quantitative analysis of data was done using fidelity level, informant consensus factor, and percent respondent knowledge indices.

**Results:**

A total of 136 medicinal plants were recorded. The plant species classified into 55 families were grouped under 14 medical categories with the highest number of plant species being used for digestive disorders (44%), followed by respiratory (38%) and dermatological disorders (36%). *Hoslundia opposita* Vahl was mentioned by 71% of the respondents for treating 22 disease conditions. Plant Family Fabaceae was the most represented with 16 species. Informant consensus agreement was high (0.7) for respiratory disorders. The fidelity level was 100% for *Bidens pilosa* L. and *Callistemon citrinus* Skeels for treating wounds and cough, respectively. Plant remedies were mainly prepared by decoction (31%) and administered orally (36%). A large number of plants (61%) were harvested from wild habitats. Herbs (50%) and leaves (50%) contributed the highest percentage of plant biological forms and parts used in remedy preparation.

**Conclusion:**

This study recorded plant species with the potential to treat a wide range of illnesses. This is reflected in the high diversity of the recorded species used for medicinal purposes. Pharmacological studies on the plants with high percentage use values and fidelity levels are needed to validate their uses in the management of the said therapeutic applications. Further research on the isolation and characterization of the plant active compounds could lead to the discovery of new potential drugs.

**Supplementary Information:**

The online version contains supplementary material available at 10.1186/s41182-021-00341-z.

## Introduction

Plants increasingly continue to form the basis of primary health care in many parts of the world [1, 2]. In many developing countries, a large proportion of the population relies on traditional medicine to meet their primary health care needs [2]. In Africa, traditional medicine has been part of the peoples’ culture, and indigenous knowledge of medicinal plants is a source of new ideas for modern pharmaceutical science [3, 4]. Likewise, in Uganda, phytotherapy still maintains an important role in meeting the primary health care needs of more than 80% of the population [5–7]. Research shows that many people take a wide range of natural products in addition to the conventional therapeutic products to manage various ailments [5, 8–14]. The increasing population of Uganda at more than 40 million makes people vulnerable to diseases and infections, due to congestion in many areas. This makes herbal medicine a better and cheaper alternative source of primary health care, especially in rural areas where modern medical services are scarce and expensive to the low-income earners. The increasing population also leads to the search for settlement land, leading to encroachment on forest resources, hence making the herbal medicines vulnerable to lose. Currently, the Uganda government has specifically upscaled the use of herbal medicine and is in the process of integrating it into the mainstream health care system [15].

The purpose of this study was to document medicinal plant uses and associated indigenous knowledge for the management of various ailments among different population groups living adjacent to Mabira and Mpanga forest reserves in central Uganda. The recorded data on respondents’ age, gender, and knowledge transfer was analyzed. This is part of an initiative to document data for future phytochemical and pharmacological studies which can act as a starting point for future discovery of drugs for various ailments. The research also contributes to the conservation and preservation of medicinal plants and traditional knowledge. Documenting medicinal plant species may also help to preserve indigenous people’s cultural heritage for future generations, since it has been passed on orally for generations by elders [16].

## Material and methods

### Study area

**Fig. 1:**
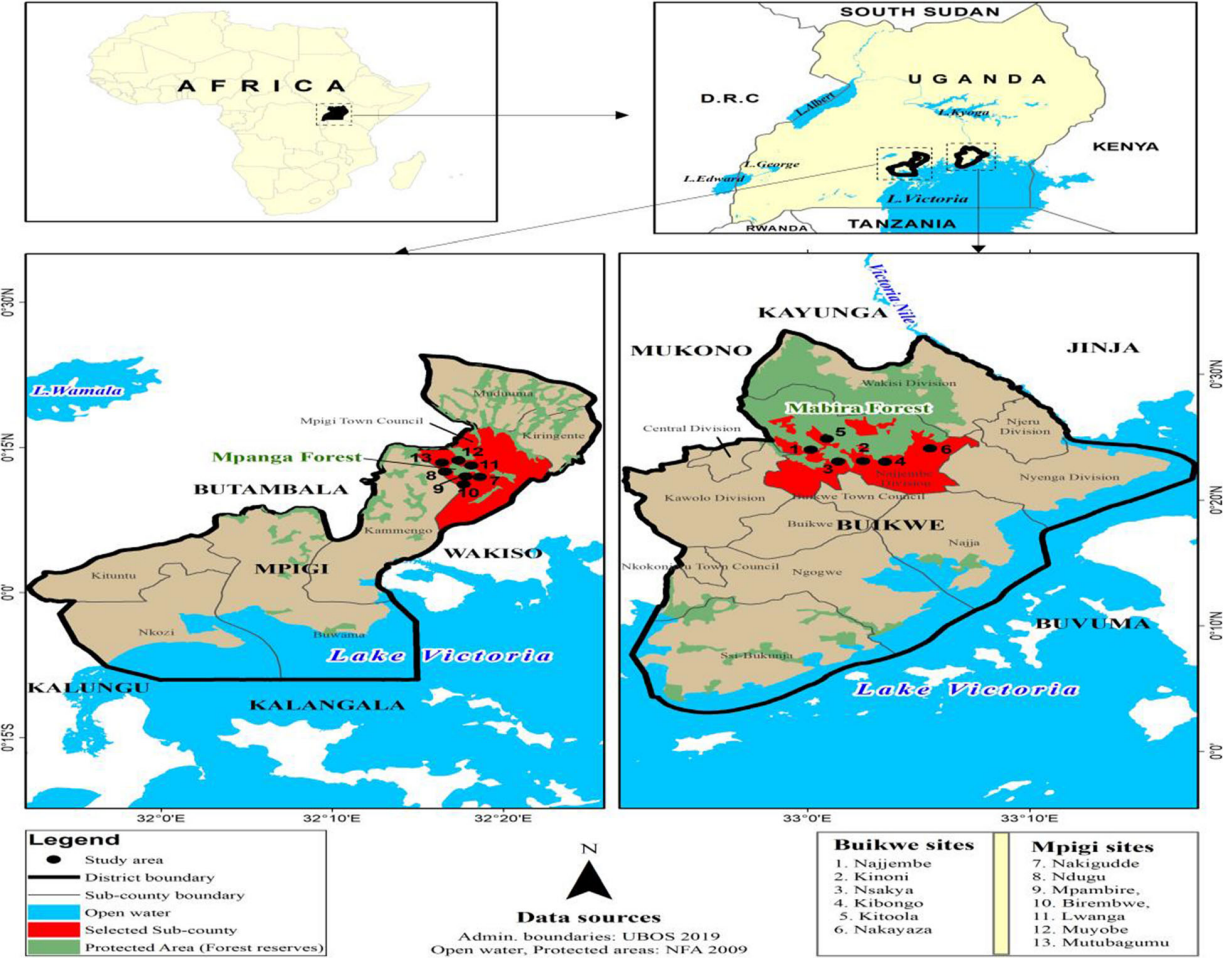
Geographical location of Study areas

This study was conducted in 7 villages adjacent to Mpanga and 6 villages adjacent to Mabira central forest reserves (CFR) (Fig. 1). The villages were within a distance of less than 5 km from the forest, hence enabling people to harvest forest resources. Mpanga forest reserve is situated in the Mpigi District about 37 km from Kampala city. Mpanga forest is protected as a scientific research site and an ecotourism center that offers a wide range of tourism activities including bird watching and forest hiking. It is one of the smallest natural equatorial rainforests with unique tree species for making drums. The forest reserve borders with districts of Wakiso to the North and East, Mityana to the North West, Butambala to the West, Kalangala to the South, and Kalungu to the South West. The site is endowed with about 500 species of trees and shrubs [17]. The dominant tree species in the Mpanga forest is the hard and weather-resistant *Celtis mildbraedii* Engl and *Bosqueia phoberos* Baill [18]. Mpanga is surrounded by a community of Baganda, whose main activity is drum making [17]. Mpigi District, often referred to as Buganda Region, is situated in the Central Region of the country and lies between latitudes 0.2^°^ South and 0.4^°^ North and longitudes 31.8^°^ East and 32.3^°^ East, with an average altitude of 1100–1400 m above sea level. The district has a bi-modal rainfall pattern (March–May & September – November) with an average rainfall amount of 1320 mm, average annual maximum temperatures range between 22.5^o^C and 27^o^C, and average relative humidity between 80% and 95% especially in forest areas. Mpigi District has 40 health units of different categories, many of which have insufficient basic equipment like a microscope, stethoscope, and medicine to offer proper health care services [19]. Because of the cross-cutting nature of health issues, there is a need for an integrated approach to health [20]. The communities however do still depend on the forest for firewood, charcoal, and medicinal herbs [17].

Mabira forest reserve is located along the main Kampala - Jinja highway, in Buikwe District. The mean annual temperature is 21°C–25°C, minimum of 16–17°C, and maximum of 28–29°C [17]. The vegetation of Mabira CFR was classified as a “medium altitude moist semi-deciduous forest with a natural habitat of 312 indigenous tree species of which *Caesalpinia volkensii* Harms is endemic [21].” Mabira forest is an important area for ecological and environmental conservation of biodiversity and habitat to many animal and plant species. The forest reserve has tea and sugarcane plantations surrounding it, where some local people are engaged as laborers. Local communities grow food crops like maize, beans, bananas, groundnuts, sweet potatoes, and vegetables mainly for subsistence consumption. However, in 2007, the government of Uganda was determined to give away up to 7000 ha of Mabira forest to Mehta to expand his sugar estate [22]. This was after the sugar prices tripled, and the Uganda government took the opportunity to try to convince the public that the only way to bring down prices was to increase sugar production by giving away part of the Mabira forest to the Sugar Corporation of Uganda Limited (SCOUL) to produce more sugar. But this proposal met stiff resistance from civil society and environmental activists who were committed to save the Mabira forest because of its rich biodiversity [22].

### Data collection and selection of study participants

Fieldwork for this study was conducted between April and June 2018. The key respondents who were mainly herbalists and community elders were selected using purposive and snowball sampling methods [23]. The respondents were selected from each parish and village on the basis of their reputation and ability to demonstrate good traditional herbal medicine knowledge. For each respondent, we recorded personal information on gender, age, and marital status. We also recorded information on the respondent’s location, the level of education, and how they acquired knowledge about medicinal plants. Before the interviews started, voluntary verbal prior informed consent of each of the informants was obtained. Since the knowledge is a natural wealth of the local people, they were assured that the data would be used only for academic purposes. After explaining the purpose of our study, we recorded information on medicinal plants and their use, plant parts used, diseases treated, the preparation and administration methods, and the conservation status (availability of the plants). Ethnobotanical data were obtained by means of semi-structured interviews and questionnaires based on standard ethnobotanical methods [24, 25]. Interviewees were also asked for the source of their knowledge in order to eliminate the information of secondary nature. Interviews were conducted in Luganda local language. The international plant name index (www.ipni.org) and the Royal Botanic Garden Kew (www.theplantlist.org) were used to validate plant scientific names, families, and authorities. Voucher specimens were identified by comparing with herbarium specimens at the National herbarium at Makerere University, Kampala, Uganda.

### Quantitative analysis of ethnobotanical data

Data were entered in an Excel sheet, and frequencies and percentages were used to summarize ethnobotanical data. These parameters were used to check for informant consensus factor (ICF), fidelity level (FL), and percentage respond knowledge (PRK) [26]. In addition, validation and homogeneity of the collected information were done by using ICF and PRK while fidelity level was used to get the percentage of respondents claiming to use a particular plant species for the same major purpose. These indices are also applied to select potential plant species for further pharmacological studies and recommendation in drug development [27].

#### Informant consensus factor (ICF)

Informant consensus factor (ICF) for different ailment categories was calculated for testing homogeneity or consistency of the informants’ knowledge about a particular remedy for a particular ailment. It is used to highlight plants of cultural relevance and agreement in the use of plants [28, 29]. The index is calculated as follows: ICF= N_ur_ – N_t_ **/** N_ur_ – 1 where N_ur_ is the number of useful reports in each category and N_t_ is the number of species (taxa) in each category. The value of this factor ranges from 0 to 1. A high ICF value indicates an agreement among respondents in the use of taxa within a medicinal category. The relative importance of a species is evaluated by the proportion of respondents who cited it.

#### Percentage of respondent knowledge (percent use-value)

The percentage of respondents who have knowledge (PRK) regarding the use of a species in the treatment of diseases was estimated using the formula:

$$ \frac{\mathrm{Number}\ \mathrm{of}\ \mathrm{people}\ \mathrm{interviewed}\ \mathrm{citing}\ \mathrm{the}\ \mathrm{species}}{\mathrm{Total}\ \mathrm{number}\ \mathrm{of}\ \mathrm{respondents}\ \mathrm{interviewed}}\times 100 $$

The percent use-value index determines the relative importance of plant species as a medicinal plant [30–34]. High PRK indicates high use reports for a plant implying its relative importance to the local community for health care needs**.**

#### Fidelity level (FL)

Fidelity level (FL) is the percentage of respondents who mention the use(s) of a certain plant species to treat a particular ailment. It indicates the respondents’ choice for a potential plant species to treat a given ailment [27, 34, 35] and is calculated using the formula: FL (%) = Np/N × 100 where Np is the number of informants that claimed use of a particular plant species for a particular disease and N is the total number of informants citing the species for any disease. The maximum FL indicates the frequency and high use of the species for treating a particular ailment by the informants in the study area. FL is designed to quantify the importance of a species for a given purpose.

#### Rahman’s similarity index (RSI)

This similarity index was used to find out the similarities and differences in traditional medicine knowledge in different study areas [36]. The similarity index shows cultural similarities between ethnic groups in the study areas by calculating particular plant species and same medicinal usage. The percentage of common uses between two study areas can be obtained using the formula [37].

$$ \mathrm{RSI}=\frac{\mathrm{d}}{\mathrm{a}+\mathrm{b}+\mathrm{c}-\mathrm{d}}\times 100 $$

where a is the number of species unique in an area A (Mpanga), b is the number of species unique in an area B, c is the number of common species in both A and B, and d is the number of common species used for similar ailments in both areas.

## Results

### Respondent characteristics

Among the 28 respondents interviewed in this study, 19 (68%) were women and the rest were men. Respondents had low education levels whereby 22 respondents (79%) out of 28 had not attended any formal education. Respondents obtained plant knowledge from their parents, grandparents, and fellow herbalists. The majority (71%) of respondents were above 40 years of age (Table 1). Ninety-six percent (96%) of respondents were married.

**Table 1:** Demographic profile of respondents (*n*=28)

Variable	Categories	Count	Percentage	Variable	Categories	Count	Percentage
**Gender**	Female	19	68	**Marital status**	Married	27	96
	Male	9	32		Widow	1	04
**Age**	20–29	3	11	**Education**	Secondary	4	07
	30–39	5	18		Primary	2	14
	>40	20	71		None	22	79

#### Medicinal plant uses and conservation of the plant species

A total of 136 medicinal plant species belonging to 55 families and 119 genera were reported to treat 57 disease conditions in both study areas. Mpanga and Mabira communities recorded 173 and 90 medicinal plant species, respectively, where 48 were common to both areas, and 24 plants had similar uses. The most abundant taxa were reported for Fabaceae (11%) and Asteraceae (9%) families. Plant species were found to be used in the management of more than one ailment, for instance, *Hoslundia opposita* Vahl (22 conditions) and *Piptadeniastrum africanum* (Hook.f) Vahl (15 conditions) (Table 2). Some similar species were found in both forests and used to treat the same ailments, for instance, *Psorospermum febrifugum* Spach, *Dracaena steudneri* Engl, and *Centella asiatica* (L.) Urb was commonly used by communities from both areas (Appendix 1). However, some plants were unique to each of the forest reserves, for instance, *Croton macrostachyus* Hochst. Ex Delile and *Abrus canescens* Baker were only found in the Mabira forest reserve. It was also noted that females recorded the highest number of plants in both areas (123 for Mpanga and 56 for Mabira) while males recorded 50 plant species from Mpanga and 34 from Mabira. Similarly, in the Mpanga forest, we recorded 56 and 24 diseases to be treated by women and men, respectively. In Mabira, there were 31 and 57 diseases treated by females and males, respectively.

**Table 2:** Ten most frequently reported diseases and species with high PRK

S/N	Plant species	Number of diseases	PRK
1	*Hoslundia opposita* Vahl.	22	71
2	*Mangifera indica* L.	4	57
3	*Momordica foetida* Schumach.	13	57
4	*Bryophyllum pinnatum* (Lam.) Oken	18	54
5	*Piptadeniastrum africanum* (Hook.f) Brenan	15	50
6	*Dracaena steudneri* Engl.	12	46
7	*Erythrina abyssinica* DC.	10	46
8	*Bidens pilosa* L.	7	46
9	*Tetradenia riparia* (Hochst.) Codd	7	43
10	*Mstroxylon aethiopicum* Sprague.	14	36

**Fig. 2:**
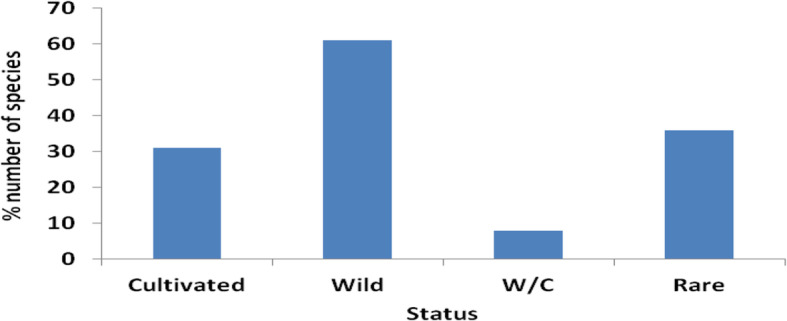
Conservation of the medicinal plant species

Throughout the study areas, 61% of medicinal plants grew in their natural (wild) environments (Fig. 2).

Wild habitats included forests, bushes, open land, and fallow land. Forty-four plant species (36%) were not easy to get from the natural environment, hence considered rare and threatened, while 10 plants (8%) occurred both in natural and cultivated habitats.

**Fig. 3:**
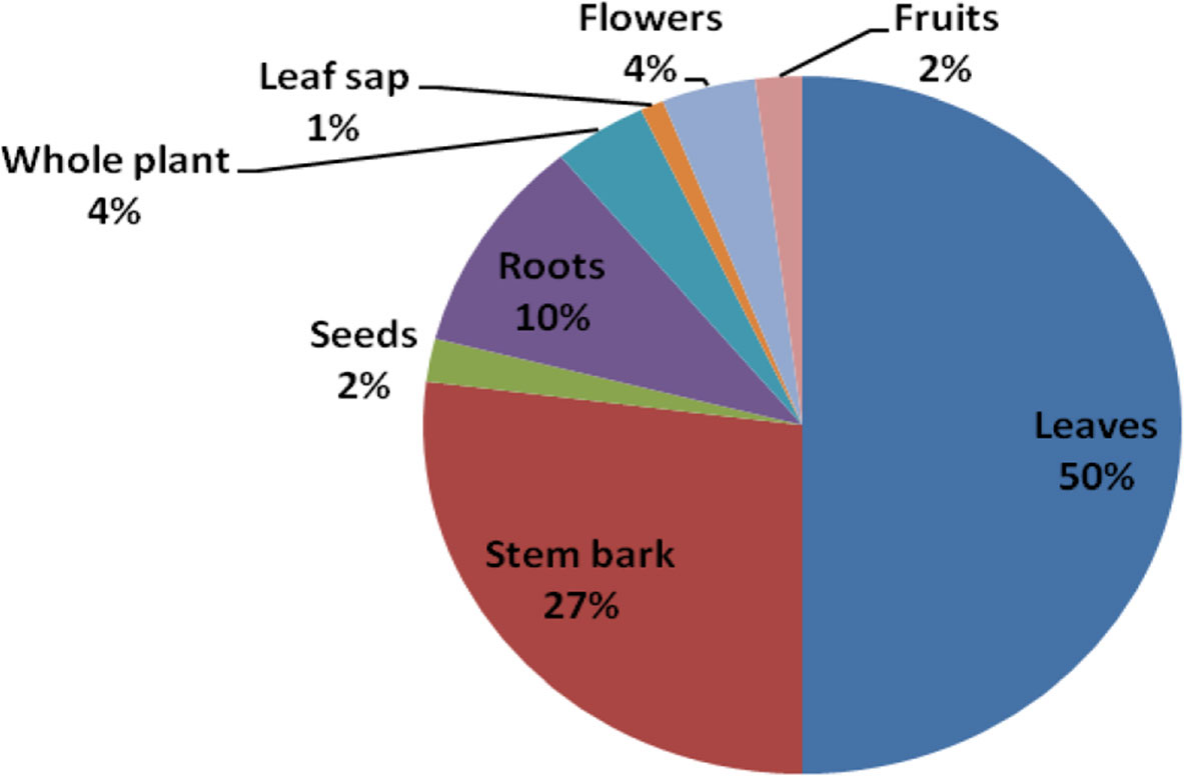
Share (%) in use reports of organs harvested

Different plant parts and biological forms were used in herbal medicine preparations (Table 2). Leaves contributed 50% of plant parts used to prepare herbal remedies (Fig. 3). However, for most plants, more than one plant part was used.

**Fig. 4:**
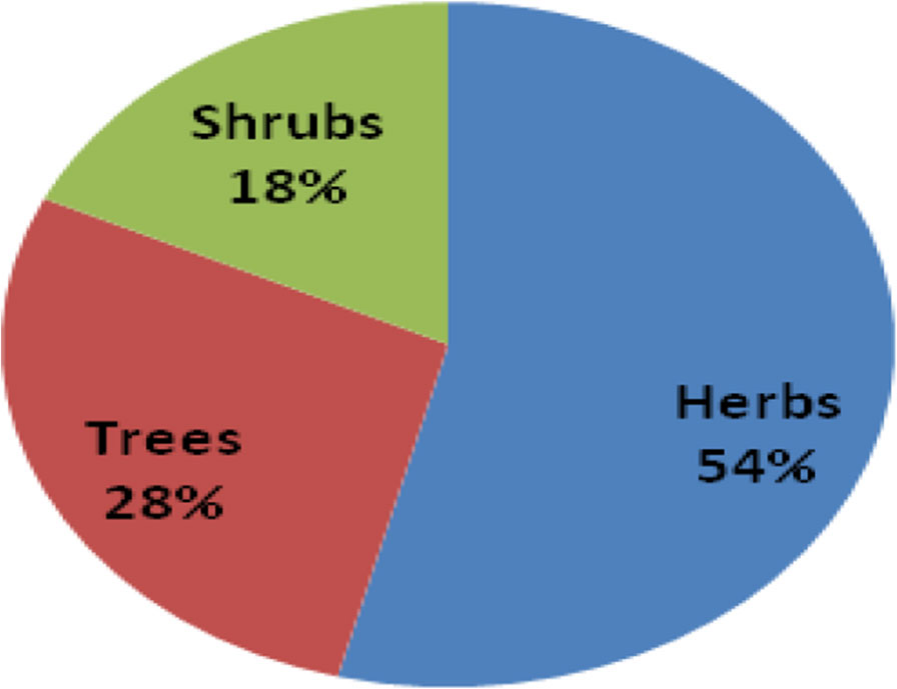
Share (%) in medicinal plant use reports of biological forms

Herbs contributed 54% of biological forms used in herbal remedy preparation (Fig. 4)

#### Herbal remedy preparation and administration

**Fig. 5:**
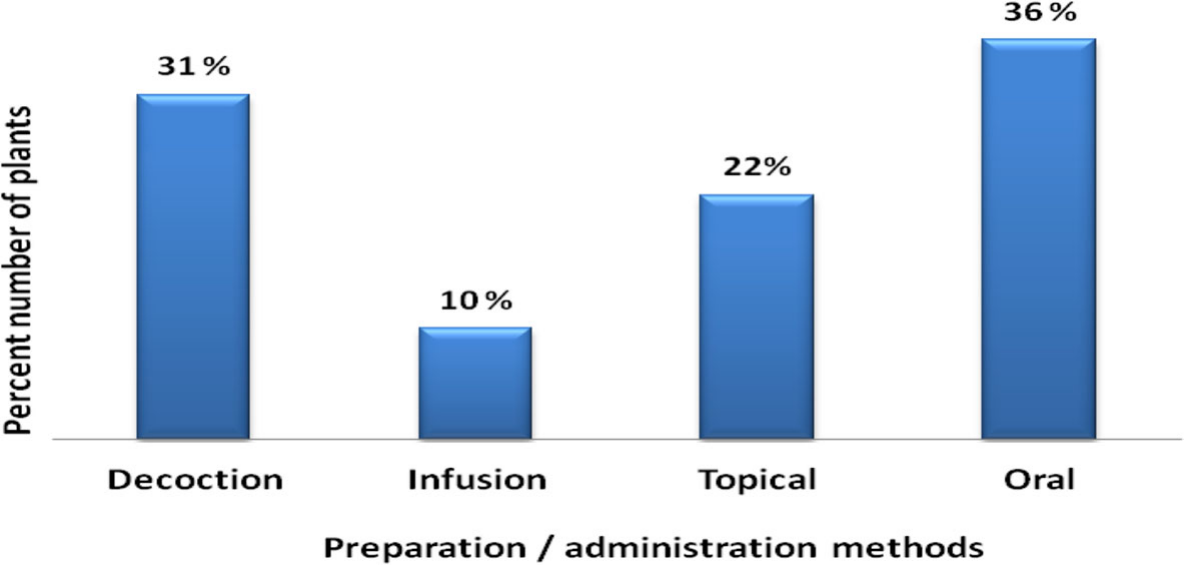
Percentage of species prepared using different methods

**Fig. 6:**
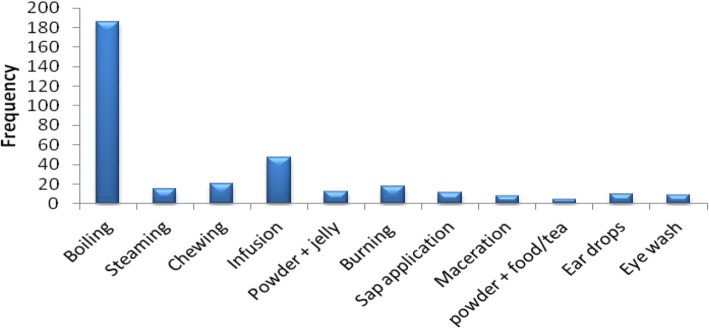
Methods of preparing herbal remedies

The most common methods of preparation and administration of medicinal drugs were decoction (31%) and oral intake (36%) respectively (Fig. 5 and Fig. 6).

#### Percentage respondents’ knowledge (percentage use-value)

**Fig. 7:**
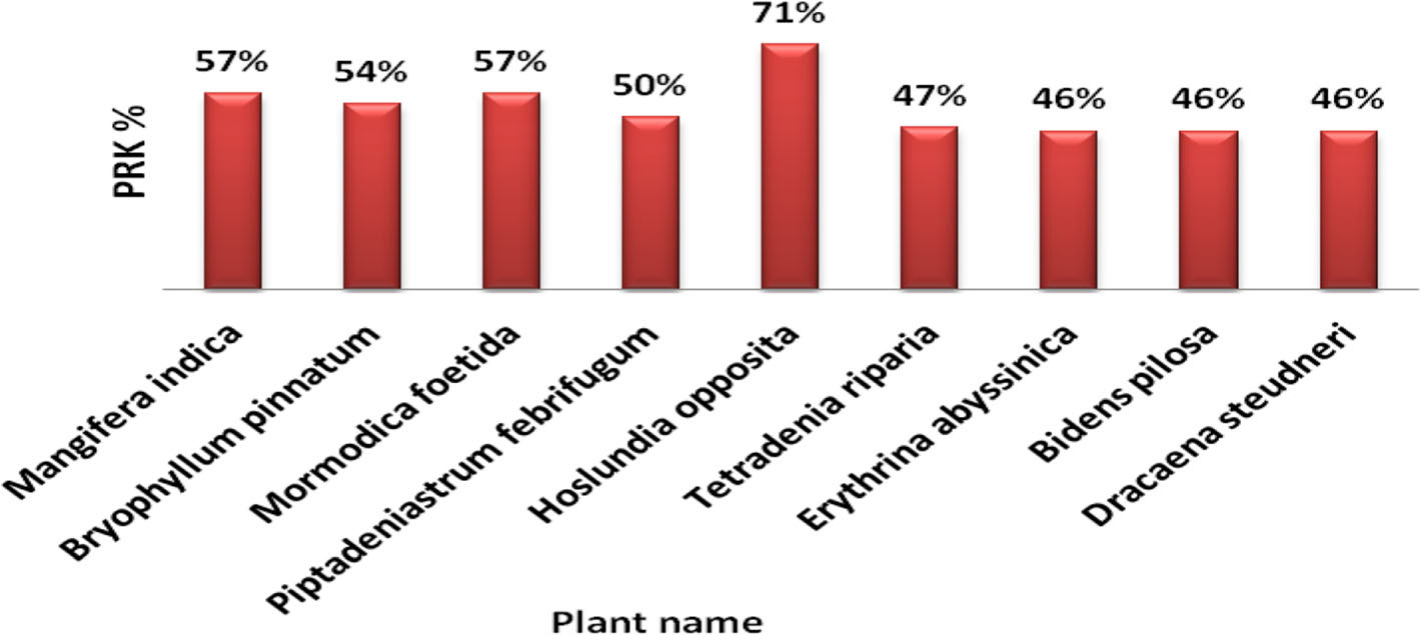
Ranking of most important medicinal plant species according to PRK

Figure 7 shows plants that were mentioned by more than 40% of respondents. The highest cultural importance was calculated for *Hoslundia opposita* Vahl. having the highest percentage (71 %) of mention by respondents.

#### Informants’ knowledge and consensus about medicinal plants (ICF)

A total of 57 disease conditions were recorded and grouped into 14 use categories (Table 3). The category of digestive disorders presented the highest number of diseases (16%) as well as plant species used (44 %). This was followed by respiratory system disorders and dermatological with 38% and 36% of plant species, respectively. Informant consensus factor (ICF) was calculated for each disease category and respiratory system disorders had the highest ICF of 0.7.

**Table 3:** Ailment categories treated by different medicinal plants

Use category	Ailments	Use citation	Number of species used	ICF
Cancers	Cancer and tumors	16	13	0.15
Cardiovascular disorders	Anemia, high blood pressure, palpitations, cleansing blood vessels	35	25	0.32
Dermatological disorders	Wounds, inflammation, skin rash, boils, warts, athletes’ foot, Paronychia	120	50	0.60
Gastrointestinal/digestive disorders	Stomachaches, ulcers, colic pain, vomiting, deworming, diarrhea, appetite boosting, dysentery, constipation	103	60	0.42
Ear, nose, and throat	Ear infections, sore throat, nose bleeding	38	26	0.32
Infectious & parasitic diseases	Malaria, headache, measles, brucella, jaundice, migraine, dizziness, hernia	48	29	0.40
Metabolic disorders	Diabetes, dehydration	6	5	0.20
Muscular and joint disorders	Chest pain, back pain, arthritis, broken & painful bones, neck pain,’	18	16	0.12
Nervous system disorders	Meningitis, convulsions, memory boost, mental illness and paralysis	16	15	0.1
Opthalmia	Itching eyes, cataract, eye infections	29	16	0.50
Reproductive health care	Blocked fallopian tubes, sexual dysfunction, antenatal care, miscarriages, barrenness, sexual dysfunction, fibroids	35	26	0.26
Respiratory system disorders	Cough, asthma, tuberculosis, sinuses	171	53	0.70
Sexually transmitted infections (STISs)	Syphilis, gonorrhea	76	40	0.50
Other purposes	Blessings, good luck, witchcraft, septic arthritis, Splenomegally	20	16	0.2

Key: Column 5: *ICF* informant consensus factor

#### Fidelity levels of frequently reported plant species

The highest fidelity level (FL: 100%) was from *Bidens pilosa* L. for wounds and from *Callistemon citrinus* (Curtis) Skeels for cough. The fidelity level for the rest of the frequently reported plants ranged from 47 to 80% (Table 4).

**Table 4:** Fidelity levels of the frequently reported plants and their major uses

Plant species	Family	Therapeutic use	N_**p**_	N	FL (%)
*Bidens Pilosa* L.	Asteraceae	Wounds	7	7	100
*Erythrina abyssinica* DC	Fabaceae	Cough	10	16	63
*Mangifera indica* L.	Anacardiaceae	Cough	16	20	80
*Psidium guajava* L.	Myrtaceae	Cough	6	10	60
*Callistemon citrinus* (Curtis) Skeels	Myrtaceae	Cough	4	4	100
*Vernonia cinerea* (L)Less	Asteraceae	Sore throat	4	7	57
*Psorospermum febrifugum* Spach	Clusiaceae	Skin infections	4	7	57
*Entada abyssinica* A. Rich	Fabaceae	Skin infections	4	6	67
*Dracaena steudneri* Engl.	Dracaenaceae	Syphilis	7	15	47

Key*:* Column 4: Np- number of respondents who use a species for a specific ailment

Column 5: N- total number of informants who mentioned the plant for any other use

Column 6: *FL* fidelity level

#### Rahman’s similarity index (RSI)

Using Rahman’s similarity index, the study shows 8.4% similarity between ethnic communities of Mabira and Mpanga where 24 plant species were common in medicinal usage to both study areas.

## Discussion

The use of medicinal plants in and around Mabira and Mpanga forest reserves is similar to many parts of the country. As seen in other communities, traditional healing is practiced by both men and women [5, 29]. The transfer of knowledge of medicinal plants from one generation to another that was mentioned in this study had been noted also by other researchers [5, 30]. It is also common practice for children and grandchildren to accompany their parents during the harvesting of medicinal plants and at the time of treating patients. This is how indigenous knowledge is acquired through time as it is handed down from one generation to another through transfer from parents to children and friends.

The dominance of some plant species in both forests could be due to the similar geographical and climatic conditions. From the results of Rahman’s similarity index (RSI = 8.4%), the study shows that there is low ethnocultural similarity in the use of medicinal plants between the two communities. However, Mpanga recorded the highest number of plant species. The plants recorded in this study have been found to be used else elsewhere in other studies for similar or different ailments. For instance, in a study done in other villages adjacent to Mabira forest reserve [31], Entada *abyssinica* A. Rich was reported for skin infections and wounds; *Oxygonum sinuatum* (Hochst & Steud ex Meisn) Dammer for boils; *Callistemon citrinus* (Curtis) Skeels for cough; and *Rhus vulgaris* Meikle for skin infections, among others. This shows the cultural importance of these plants to these communities.

In our study, the predominance of Fabaceae, Asteraceae, and Lamiaceae families in medicinal use is not new as studies from other researchers report similar findings [7, 9, 11, 32, 33, 35, 38, 39]. Fabaceae is the third-largest family and is of great ethnobotanical importance in indigenous and urban communities throughout the world [40]. The family Fabaceae has also been reported in other studies to treat anemia, diarrhea, and cancer [41]. The therapeutic properties of Fabaceae are attributed to the presence of flavonoids (the main constituents), tannins, saponins, alkaloids, and terpenes which are known to possess high levels of bioactivity. The active compounds from this family possess antibacterial, antioxidant, and anti-fungal activities [41]. This may justify their use in the treatment of skin infections, cough, ear infections, cancer, wounds, and syphilis as reported in this study.

The predominance of harvesting plants from the wild has been observed in other studies where over 100 different types of medicines were collected from natural forests [42]. This puts the key plant species at risk of disappearing since they are threatened by human activities like agriculture and seasonal variations. This study recorded *Milicia excelsa* (Welw) C.C. Berg as threatened. This species also appears under the Red List of threatened species of Uganda 2018 [43].

The use of different plant parts ranging from leaves, roots, fruits, seeds, and whole plants for herbal remedy preparation has been recorded by other researchers [8–11, 44]. The predominance of using leaves, biological form (herbs), mode of preparation (decoction), and administration (oral intake) methods has been observed in earlier ethnobotanical studies [7, 11, 19, 31, 45–47]. The predominance in the use of leaves could be due to their fast regeneration [11] and the photosynthetic and biosynthetic activities which lead to the production of most bioactive substances [8].

The use of plant mixtures for herbal remedy preparation was also reported in other studies [48], and this could be due to the additive or synergistic effects of the combined plant compounds that act on different pathogens.

The highest frequency of mention of *Hoslundia opposita* Vahl for treating various ailments has also been reported elsewhere [49]. This could be due to the numerous pharmacological compounds and properties that the plant contains [49].

The highest ICF (0.71) which was recorded for respiratory disorders has been reported to be the dominant disease category in other study areas [46, 47, 5, 4, 51, 11].

*Bidens pilosa* and *Callistemon citrinus* had a fidelity level of 100% and ranked highest in treatment of wounds and cough, respectively. The potential of these plants to treat the same ailments has been reported by other authors [7, 11, 14, 41, 49–54].

## Conclusion

The people in and around Mabira and Mpanga forest reserves widely use medicinal plants to manage various human ailments. This shows that the preservation and conservation of indigenous knowledge are vital for the sustainable utilization of the plant resources. There is a need for immediate conservation of the threatened and disappearing species to avoid their extinction from the wild. Plants with high informant agreement and fidelity level values can be subjected to further pharmacological studies to validate their traditional uses. This can also lead to the discovery of new bioactive molecules. The reported ethnobotanical studies by other researchers validate the use of the recorded plants in the treatment of the mentioned ailments, although further investigations need to be done in areas of pharmacology and toxicology.

## Supplementary Information

**Additional file 1: Appendix 1.** Ethnobotanical data on the medicinal plant species used in the study areas.

## Data Availability

All data generated during the survey and analyzed is available on request from the corresponding author.
