# Supplementary material for: Ethnobotanical survey of medicinal plant species used by communities around Mabira and Mpanga Central Forest Reserves, Uganda
Source: Trop Med Health. 2021 Jun 29;49:52. doi: 10.1186/s41182-021-00341-z (PMC8243914; doi:10.1186/s41182-021-00341-z)
Supplement: Supplementary file 1 — Additional file 1: Appendix 1. Ethnobotanical data on the medicinal plant species used in the study areas. [file 41182_2021_341_MOESM1_ESM.doc]

**Appendix 1**. Ethnobotanical data on the medicinal plant species used in the study areas

| **Plant Family & Scientific name** | **Local name (Luganda)**  **& code number** | **Growth form** | **Parts used** | **Ailments** | **preparation & application** | **Status** | **PRK**  **(n=28)** | | **Literature reports** |
| --- | --- | --- | --- | --- | --- | --- | --- | --- | --- |
| **Acanthaceae** *Dicliptera laxata* C. B. Clarke | Muzukizi (AS046) | Herb | L, Bk | Anaemia (2); Convulsions | Decoction drunk | **C/R** | 10.7 | | Antidote (Segawa & Kasenene, 2007) |
| *Justicia betonica* L. | Nalongo (AS097) | Herb | L | Meningitis | Squeeze, drink cold extract | **C/R** | 10.7 | | Fever, Fungal infections |
|  |  |  | L | Malaria (2) | Decoction drunk |  | 7.1 | | (Nambejja et al, 2019) |
| *Justicia engleriana* Lindau | Omuwanga (AS121) | Shrub | L | Malaria | Decoction drunk | **W/R** | 3.5 | | no literature |
| *Justicia exigua* S. Moore | Kazunzanjuki (AS122) | Herb | Wp | Dizziness | Macerate and bathe | **W/R** | 3.5 | | Salpingitis (Tabuti et al, 2003) |
| **Amaranthaceae**  *Aerva lanata* (L.) Juss | Olweza (AS124) | Herb | L | Skin infections | Decoction bathed | **W/A** | 7.1 | | Segawa & Kasenene, 2007 |
|  |  |  | Wp | Good luck charm | Decoction bathed | |  | | Body odur (Tugume et al, 2016) |
| *Chenopodium opulifolium* Schrad. ex W.D.J.Koch & Ziz | Omwetango (AS146) | Herb | L | Measles | Mixed with *Phyllanthus capillaris*, decoction drunk | **C/R** | 21.4 | | Malaria (Namukobe et al, 2011) |
|  | |  | L | Sore throat (4) | Chewed with salt, | |  | |  |
|  |  |  | L | Cough | Infusion drunk | |  | |  |
|  |  |  | L | Sinus (2) | Pounded fresh with rock salt, warmed in  clean cloth, extract drunk | | | | |
| *Chenopodium procerum* | Omugosola (AS147) | Herb | L | Malaria | Infusion drunk | **W/C/A** | 3.5 | | Muscle pains, headache |
| Hochst. Ex Moq. | |  |  |  |  |  |  | | (Namukobe et al 2011) |
|  | |  |  |  |  |  |  | |  |
| **Anacardiaceae** *Mangifera indica* L | Emiyembe (AS025) | Tree | L | Skin infections | Leaf sap applied | **C/A** | 57 | | Diarrhoea, cough (Appiah t al, 2018) |
|  |  |  | L, Fr | High blood pressure | Decoction drunk or fruit eaten | | | |  |
|  |  |  | L | Ulcers (2) | Macerate powder to hot water, drink | | | |  |
|  |  |  | L, Bk, Fr | Cough (16) | Powder chewed with salt or boiled | | | | |
| *Rhus natalensis* Bernh.ex. C. Krauss | Akakwansokwanso akatono | Shrub | L | Jaundice, Pressure | Decoction drunk | **W/R** | 14.3 | | HIV/AIDS care (Kisangau et al, 2011) |
|  | (AS067) |  | L | Wounds | Decoction drunk/bathed | |  | | Digestive disorders (Asiimwe et al, 2013) |
|  |  |  | L | Skin infections | Powdered jelly applied on skin | | | | |
| *Rhus vulgaris* Meikle | Akakwansokwanso akanene | Shrub | L | Blood plasma , Fever | Decoction drunk | **W/R** | 14.3 | Cough, jaundice, GIT | |
|  | (AS042) |  | L | Skin infections, syphilis | Decoction bathed |  |  | Syphilis, malaria (Okullo et al, 2014) | |
| **Annonaceae** *Annona muricata* L. | Ekitaferi (AS125) | Tree | Fr, L | HBP, Ulcers | Leaf decoction drunk or fruits eaten | **C/R** | 7.1 | HBP (Leonard et al., 2016), stomachache, dizziness (Tantengco et al, 2008) | |
| **Apiaceae**  *Centella asiatica (*L.) Urb | Kabo kamuwala /embutamu | Creeping herb | L | Cough (4) | Decoction drunk | **W/A** | 39.3 | TB (Bunalema et al, 2013) | |
|  | (AS126) |  | Sap | Wounds | Sap applied |  |  | Neuroprotective (Orhan, 2012) | |
|  |  |  | L | Itching eyes | Eyes washed with extract | |  | wounds, antioxidant, anticancer, | |
|  |  |  | L | Cancer, Fallopian tubes | Decoction drunk | |  | Antidiabetic, Prakash et al, 2012) | |
|  |  |  | L | Diarrhoea (2) | Infusion drunk |  |  |  | |
|  |  |  | L | Antenatal care | Infusion drunk | | |  | |
| *Steganotaenia araliacea* Hochst. | Muwanula (AS115) | Herb | L | Removes curses | Infusion drunk | **C/A** | 3.5 | TB (Bunalema et al, 2013; convulsions, labour pains (Asiimwe et al, 2014) | |
|  |  |  |  |  |  |  |  |  | |
| **Apocynaceae** *Alstonia boonei* De Willd | Mubajangalabi (AS127) | Tree | Bk | Back pain, Syphilis, Fallopian tubes | Decoction drunk | **W/A** | 14.3 | Malaria (Adebayo & Krettli, 2011) | |
|  |  |  |  | Stomach infections | |  |  |  | |
| *Mondia whitei* (Hook.f) Skeels | Mulondo (AS128) | Climbing herb | R | Aphrodisiac | Chew | **W/A** | 3.5 | Aphrodisiac (Ssozi et al, 2016; | |
|  |  |  |  |  |  |  |  | Namukobe et al, 2011) | |
| *Secamone africana* (Oliv.) Bullock | Akatakura (AS129) | Herb | Fl, L | Ear infections | Steam in banana leaves, drop extract in ear | **W/A** | 7.1 | Constipation, malaria (Namukobe et al, 2011) | |
|  |  |  | Fl, L | Constipation | Decoction drunk | |  |  | |
| *Thevetia peruviana* (Pers) K. Schum. | Kasenene (AS022) | Tree | sap | Warts | leaf sap applied | **C/A** | 3.5 | Cough/TB (Asiimwe et al, 2013) | |
| **Arecaceae**  *Serenoa repens* (W. Bartram) Small | Empirivuma (AS130) | Tree | Bk | HBP, painful neck | Powder decoction drunk | **W/R** | 3.5 | Prostatic hypertrophy (Baenes et al, 2007) | |
| *Phoenix reclinata* Jacq. | Ekisansa /ekikindukindu (AS186) | Shrub | R | Cough & other respiratory disorders | 5ml of decoction drunk by adults; 1 TS child daily) | **W/R** | 3.5 | Hernia (Kibuuka & Anywar, 2015) | |
| **Aristolochiaceae** *Aristolochia elegans* Mast | Lukaawa (AS131) | Creeping herb | seeds | Malaria (2) | Seeds chewed or decoction drunk | **W/C/A** | 7.1 | Tugume at al, 2016; Bunalema et al, 2013 | |
| **Asclepiadaceae** *Gomphocarpus physocarpus* E. Mey | Akafumbo (AS132) | Herb | L, R | Nausea (2) | Decoction drunk | **W/R** | 7.1 | Malaria (Aidia et al, 2014) | |
| **Asparagaceae** *Dracaena steudneri* Engl. | Kajjo lyanjovu (AS153) | shrub | L, Bk | Syphilis (5) | Boil & bathe or drink 500ml daily for a week | **W/A** | 46.4 | TB (Bunalema et al, 2013) | |
|  |  |  | L | Wounds (5) | Macerate in hot water, squeeze, apply | |  | HIV/AIDS care (Kisangau et al, (2011) | |
|  |  |  | L | Stomachache (2) | Macerate or boil & drink | |  |  | |
|  |  |  | L | Asthma, Pressure | Decoction drunk |  |  |  | |
|  |  |  | L | cough (2) | Ash licked |  |  |  | |
|  |  |  | L | Skin infections | Decoction bathed | |  |  | |
|  |  |  | Bk | Blocked Fallopian tubes | Decoction drunk | |  |  | |
|  |  |  | L | Ear infections | Squeeze, apply drops in ear | |  |  | |
|  |  |  | Bk | Arthritis | Decoction drunk for 9 days | | |  | |
|  |  |  | Bk | Back pain, Cancer | Decoction drunk | |  |  | |
| **Asteraceae** *Ageratum conyzoides* (L.) L. | Namirembe (AS116) | Herb | L | Anaemia | Decoction drunk | **W/A** | 3.5 | Skin infections | |
|  |  |  | L | Skin infections | Boil, bathe |  |  | (Nambejja et al, 2019 | |
| *Bidens pilosa* L. | Sere (AS102) | Herb | L | Wounds (7) | Extract applied on affected area | **W/A** | 46.4 | wounds, ulcers, | |
|  |  |  | L | Ulcers (2) | Dry decoction or infusion drunk | | | Diarrhoea, nose bleeding, | |
|  |  |  | L | Sore throat | Decoction drunk | |  | eye infection (Namukobe et al, 2011) | |
|  |  |  | L | Syphilis | Decoction drunk/bathed | |  |  | |
|  |  |  | L | Itching eyes | Eye wash with infusion | | |  | |
|  |  |  | R, L | STIs, Anaemia | Decoction drunk | |  |  | |
| *Conyza floribunda* Kunth | Kafumbe (AS133) | Herb | L | Boils | Roast and squeeze, apply extract | **W/A** | 10.7 | HIV/AIDS care (Kisangau et al, 2011) | |
|  |  |  | L | Sore throat | Steamed extract drunk | | |  | |
|  |  |  | L | Skin infections | Fresh extract smeared | |  |  | |
|  |  |  | L | Flue | Decoction drunk | |  |  | |
| *Crassocephalum vitellinum* (Benth.) S. Moore | Ekitonto (AS055) | Herb | L | Eye infection (2) | Steamed extract dropped in eyes | **C/A** | 10.7 | Syphilis (Asiimwe et al, 2014); malaria (Adia et al, 2014) | |
|  |  |  | L | Cough | Ash licked | |  |  | |
|  |  |  | L | Syphilis | Infusion drunk | | |  | |
| *Dichrocephala integrifolia* (L. f) Kuntze | Buza (AS023) | Herb | L | Appetite boosting, Deworming | Decoction drunk | **W/A** | 3.5 | Burns, diarrhea (Ssozi et al, 2016; Asiimwe et al, 2013) | |
|  |  |  | L | Boils | Leaf extract applied | |  |  | |
| *Emilia caespitosa* Oliv. | Oluwomerambuzi (AS134) | Herb | Wp | Syphilis | boil with leaves of *Vernonia cinerea* & *Hoslundia opposita,* drink & bathe | **W/A** | 3.5 | no literature | |
| *Erlangea tomentosa* (Oliv. & Hiern.)S. Moore | Etwatwa (AS056) | Herb | L | Eye infection | Decoction applied | **W/A** | 21.4 | Miscarriage, colic, fever | |
|  |  |  | L | Stomachache (2) | Decoction drunk | |  | stomachache, syphilis | |
|  |  |  | L | Post-natal care | Infusion drunk |  |  | (Asiimwe et al, 2014) | |
|  |  |  | L | Skin infections, Flue | Decoction drunk | |  |  | |
|  |  |  | L | Syphilis | Decoction drunk and bathed | | |  | |
| *Leonotis nepetifolia* (L.) R. Br | Ekifumufumu (AS135) | Herb | L | Ear infection | Steam extract applied in ears | **W/C/A** | 32.1 | Hernia (Kibuuka & Anywar, 2015) | |
|  |  |  | L | Gonorrhea | Decoction drunk | |  | Wounds (Namukobe et al, 2011) | |
|  |  |  | L | Diarrhoea (2) | Infusion drunk |  |  |  | |
|  |  |  | L | Syphilis (2) | Decoction drunk and bathed | | |  | |
|  |  |  | L | Cough & flue (2) | Infusion drunk | |  |  | |
|  |  |  | L | Pneumonia, Hernia | Decoction drunk | |  |  | |
|  |  |  | R | Chest pain | Rub the affected part | |  |  | |
| *Microglossa pyrifolia* (Lam.) Kuntze | Kafugankande (AS036) | shrub | L | Diarrhoea | Infusion drunk | **W/C/A** | 7.1 | Hernia (Kibuuka & Anywar, 2015) | |
|  |  |  | L | Cancer, Pressure | Decoction drunk | |  |  | |
|  |  |  | L | Fallopian tubes | Decoction drunk | |  |  | |
| *Sigesbeckia orientalis* L. | Seziwundu (AS136) | Herb | L | Wounds (2) | Extract applied on wounds | **W/R** | 3.5 | Restore blood circulation, STIs, sores | |
|  |  |  | L | Cough, Nose bleeding | Infusion drunk | |  | (Burkil, 1985) | |
| *Vernonia amygdalina* Delile | Mululuza (AS062) | shrub | L | Fever (2) | Infusion drunk | **W/A** | 32.1 | Typhoid, malaria | |
|  |  |  | L | Cough (2) | Infusion drunk; or powder chewed with salt | | | Diarrhoea (Appiah et al, 2018) | |
|  |  |  | L, R | Malaria (5) | Infusion drunk | |  |  | |
|  |  |  | L | Skin infections | Boil, or drink infusion; bathe | | |  | |
| *Vernonia auriculifera* Hiern | Ebikokoma (AS137) | Herb | L | Syphilis | Decoction drunk & bathed | **W/A** | 7.1 | Diarrhoea (Woldeab et al, 2018) | |
| *Vernonia cinerea* (L.) Less | Kayayana (AS138) | Herb | L | Sore throat (4) | Leaf chewed | **W/A** | 25 | malaria (Adia et al, 2014) | |
|  |  |  | L | Skin infections (2) | Mix with roots of *Solanum campylacanthum*, boil & bathe | | | Eye disease (Aravindhan & Rajendran 2013) | |
|  |  |  | L | Eye/ Ear infections | Infusion applied | | |  | |
|  |  |  | L | Wounds | Infusion applied | |  |  | |
| **Balanitaceae** *Balanites wilsoniana* Dawe & Sprague | Naligwalimu (AS139) | Herb | Bk | Mental illness, Stomachache, Gonorrhea, Syphilis | Decoction drunk | **W/R** | 3.5 | Malaria, cosmetics vegetable oil (Burkil 2009) | |
| **Bignoniaceae** *Kigelia africana* (Lam) Benth | Omussa (AS140) | Tree | Bk | Chest pain | Boil for 3 hrs, drink for 9 days | **W/R** | 10.7 | Stomachache (Appiah et al, 2018) | |
|  |  |  | Bk | Arthritis | Boil for 3 hrs, drink for 9 days | | | Cough (Ssozi et al, 2016) | |
|  |  |  | Bk | Back pain, Cancer | Boil for 3 hrs, drink for 9 days | | |  | |
|  |  |  | Bk | Cough | Pound, dry, boil + rock salt or ginger, drink | | | | |
|  |  |  | L | Skin infections | Boil and bathe |  |  | |  |
| *Markhamia lutea* (Benth) K. Schum. | Omusambya (AS070) | Tree | Bk | Cough (2) | Boil, cool, drink 3x daily | **C/A** | 14.3 | | Malaria (Adia et al, 2014) |
|  |  |  | Fl, L | Ear infections (2) | Squeeze or steam & apply drops in ear | | | | |
| *Spathodea campanulata* P. Baeuv | Ekifa bakazi (AS211) | Tree | L | Antenatal care | Infusion drunk | **C/A** | 14.3 | | Wounds, typhoid, |
|  |  |  | L | Cancer & Fallopian tubes | Decoction drunk | |  | | Malaria (Appiah et al, 2018) |
|  |  |  | Bk | Syphilis (2) | Boil & bathe, or drink 1/2 cup 2x daily | | | | Syphilis (Ssozi et al, 2016) |
|  |  |  | Bk | Stomach infections | Decoction drunk | |  | |  |
|  |  |  | Bk | Wounds | Dry, powder, apply 3 x daily | | | |  |
| **Brassicaceae** *Cardamine trichocarpa* Hochst. Ex. Rich. | Mageregankoko (AS141) | Herb | Wp | Migraine | Steam & inhale vapour | **W/R** | 3.5 | | Kwashiorkor (Jansen, 2004) |
| **Burseraceae** *Canarium schweinfurthii* Engl. | Omuwafu (AS142) | Tree | Bk | Cough (2) | Decoction drunk | **W/C/A** | 7.1 | | Anaemia, cough, GIT, diarhoea, jaundice, eye infection, deworming, (Okullo et al, 2014) |
| **Cannabaceae** *Cannabis sativa* L. | Olusambya (AS143) | Herb | Fl, L | Ear infection | Steamed extract dropped in ear | **C/R** | 7.1 | | Skin burns (Abbasi et al, 2010) |
|  |  |  | R | Diarrhoea | Decoction drunk | |  | | Measles (Tugume et al, 2016) |
| **Canellaceae** *Warburgia Ugandensis* Sprague | Barwegira (AS041) | Tree | L | Fever | Infusion drunk | **W/C/R** | 18 | | Pneumonia, asthma, |
|  |  |  | L | Sore throat | Pound, dry, boil + rock salt or ginger, drink | | | | Skin infections  Candida, Malaria (Okello & Kang, 2019) |
|  |  |  | L | Gonorrhea | Decoction drunk | |  | |
|  |  |  | Bk | Cough (2), | Bark chewed |  |  | |  |
|  |  |  | Bk | Syphilis | Decoction drunk | |  | |  |
|  |  |  | Bk | Cancer/tumors | Boil, drink 3 x for 9 days | |  | |  |
|  |  |  | Bk | Convulsions | Decoction drunk | |  | |  |
|  |  |  | Bk | Stomach infections | 250ml of decoction drunk | |  | |  |
|  |  |  | Bk | Sexual dysfunction | 250ml of decoction drunk | |  | |  |
|  |  |  | Bk | Anemia | Boil, drink 3 x for 9 days | |  | |  |
| **Caricaceae**  *Carica papaya* L. | Papari (AS024) | Tree | R | Blocked fallopian tubes | Decoction drunk | **C/A** | 3.5 | | Malaria (Appiah et al, 2017; Ngarivhumr et al, 2015) |
| **Celastraceae** *Maytenus senegalensis* (Lam) Exell | Naligwalimu (AS060) | Shrub | Bk | Mental illness, Stomachache | Decoction drunk | **W/R** | 7.1 | | TB (Bunalema et al, 2013) |
|  |  |  | Bk | Gonorrhea, Syphilis | Decoction drunk | |  | | Syphilis, asthma, herpes zoster (Asiimwe et al, 2013; 2014) |
| *Mystroxylon aethiopicum* (Thunb.)Loes | Entasesa (AS144) | Tree | L,R | Skin infections (3) | Fresh leaf decoction bathed | **W/A** | 36 | | Hemorrhagic diarhoea, stomachache, |
|  |  |  | L, Bk | Flue, cough (2) | Boil, drink 3 TS daily | |  | | respiratory infections |
|  |  |  | L | Eye infection | Boil, drop in eyes | |  | | anaemia ( Kilonzo et al, 2016) |
|  |  |  | L | Paronychia | Roast, apply on affected area | | | |  |
|  |  |  | L, Bk | Syphilis & gonorrhea (2) | Pound, add water, drink | |  | |  |
|  |  |  | Bk | Stomachache | Decoction drunk | |  | |  |
|  |  |  | Bk | Diabetes (2) | Decoction drunk | |  | |  |
|  |  |  | L | Ulcers, Pressure | Decoction drunk | |  | |  |
|  |  |  | Bk | Arthritis, cancer | Decoction drunk | |  | |  |
|  |  |  | L | Memory boost | Decoction drunk | |  | |  |
|  |  |  | Bk | Back pain | Boil for 3 hrs, drink for 9 days | | | |  |
| *Salacia elegans* Welw.ex.Oliv | Akamwanyimwanyi (AS145) | Herb | L | Boils, wounds | Squeeze, apply on affected area | **W/A** | 7.1 | | No literature |
|  |  |  | Bk | Syphilis | 500ml of decoction daily for a week | | | | |
|  |  |  | L | Dysentery | Decoction drunk | |  | |  |
|  |  |  | L | Skin infections | Boil or apply sap | |  | |  |
| **Cleomaceae**  *Cleome gynandra* L. | Ejjobyo (AS017) | Herb | L | Cough, Fever | Decoction drunk x3 daily | **C/A** | 7.1 | | HIV/AIDS care (Kisangau et al, 2011) |
| **Clusiaceae**  *Garcinia buchananii* Baker | Omusali (AS149) | Tree | Bk | Cough (2) | Pound, dry, add water, boil + rock salt or ginger, drink | **C/R** | 7.1 | | GIT, asthma, allergy, cough, eye, cardiovascular (Okullo et al, 2014) |
| **Combretaceae** *Combretum molle* Engl. & Diels | Endagi (AS105) | Tree | Bk, L | Syphilis (2) | Boil, drink and bathe | **W/R** | 28.6 | | Malaria (Adia et al, 2014) |
|  |  |  | L | Wounds (2), Skin infections | Boil or apply sap | |  | | TB (Bunalema et al, 2014) |
|  |  |  | L, Bk | Asthma, cough | Boil, drink 1/2 cup twice daily | | | |  |
|  |  |  | Bk | Blocked fallopian tubes | Decoction drunk | |  | |  |
|  |  |  | L | Gonorrhea | Dry, boil powder with water + rock salt or ginger, drink | | | | |
| **Crassulaceae** *Bryophyllum pinnatum* (Lam) Oken | Ekiyondo ekyeru (AS151) | Herb | L, Bk | Cough (4) | Boil & drink; or dry, add salt, lick | **C/A** | 54 | | Wounds, burns |
|  |  |  | L, Bk | Wounds/boils | Boil or steam & apply on skin | | | | (Nambejja et al, 2019) |
|  |  |  | L | Jaundice, Fever, Anemia | Boil or steam & drink | | | | Diarrhoea, wounds, vomiting, |
|  |  |  | L | HBP (2), sore throat | Boil or steam & drink | | | | respiratory tract infections, boils |
|  |  |  | L | Ear infection (3) | Boil or steam & drop in ear | | | | (Onoja et al., 2018; Gurnani et al, 2017) |
|  |  |  | L | Eye infection (2) | Boil or steam & drop in eyes | | | | |
|  |  |  | L | Appetite & deworming in children | Boil or steam & drink | | | |  |
|  |  |  | Bk | Back pain, Stomachache | Decoction drunk | |  | |  |
|  |  |  | Bk | Blocked fallopian tubes | Decoction drunk | |  | |  |
|  |  |  | Bk | prevent miscarriage, Nausea | Decoction drunk | |  | |  |
|  |  |  | Bk | Syphilis (4) | Decoction drunk/bathed | |  | |  |
| *Kalanchoe crenata* (Andrews) Haw. | Ekiyondo (green) (AS093) | Herb | L | Diarrhoea | Infusion drunk | **C/R** | 7.1 | | Otitis, headache, inflammations, convulsions |
|  |  |  |  | HBP | Decoction drunk | |  | | (Nguelefack et al, 2006) |
|  |  |  |  | Ear infections | Squeeze, drop extract in ear | | | |  |
| **Cucurbitaceae** *Momordica foetida* Schumach | Ebombo (AS086) | creeping herb | L, Bk | Cough (11) | Decoction with salt & ginger drunk | **W/A** | 57.1 | | Malaria (Ngarivhume et al, 2015) |
|  |  |  | Bk | Skin infections | Decoction bathed | |  | | TB (Tabuti et al, 2010) |
|  |  |  | L, Bk | Wounds (2) | Decoction bathed | |  | |  |
|  |  |  | L | Wounds in the uterus and tubes | Infusion drunk | | | | |
|  |  |  | Fl | Cleansing stomach & blood vessels | Dry, pound, drink powder with hot water | | | | |
|  |  |  | L | Syphilis, ulcers, diarrhoea | Decoction drunk | |  | |  |
|  |  |  | L | Eye infection (2) | Boil, wash eyes |  |  | |  |
|  |  |  | L | Jaundice & Fever | Mix with *Fleuggea virosa*, boil, drink | | | | |
|  |  |  | L | Nausea | Decoction drunk | |  | |  |
|  |  |  | Fl | Anaemia | Powder drunk with hot water | | | | |
| *Zehneria scabra* Sond | Kabindizi (AS034) | creeping herb | L | Wounds (2) | Decoction drunk | **W/A** | 10.7 | | Diarrhoea (Woldeab et al, 2018) |
|  |  |  | L | Wounds | Decoction drunk | |  | |  |
|  |  |  | L, Wp | Skin infections (2) | Powdered jelly used to smear body | | | | |
|  |  |  | Wp | Syphilis | Decoction drunk | |  | |  |
| **Euphobiaceae** *Croton macrostachyus* Hochst. Ex Delile | Omusogasoga (AS155) | Tree | L | HBP; diabetes | Decoction drunk | **W/R** | 3.5 | | Diarrhoea, (Woldeab et al 2018) |
| *Euphorbia hirta* L. | Akasandasanda (AS027) | Herb | L | Cough | Boil, drink 3 x daily | **W/A** | 10.7 | | wounds, cough |
|  |  |  | L | Wounds | Decoction drunk | |  | | (Appiah et al, 2018) |
|  |  |  | L | Constipation (2) | Decoction drunk | |  | | Hernia (Kibuuka & Anywar, 2015) |
| *Euphorbia enterophora* Drake | Lusanda (AS154) | Herb | L | Deworming | Decoction drunk | **W/A** | 3.5 | | no record |
| *Jatropha carcus* L. | Ekirowa (AS010) | shrub | L | Syphilis | Decoction drunk, bathed | **W/C/A** | 36 | | wounds, toothache |
|  |  |  | L, Bk | Cough (3) | Steam extract drunk | |  | |  |
|  |  |  | L | Diarrhoea | Infusion drunk |  |  | |  |
|  |  |  | L | Wounds (4) | Leaf sap applied | |  | |  |
|  |  |  | Bk | Cancer, back pain, arthritis | Boil for 3 hrs, drink for 9 days | |  | |  |
| *Sapium ellipticum* (Hochst.) Pax | Omusasa (AS158) | Tree | Bk | cough (2) | Boil, drink 3 x daily | **W/A** | 7.1 | | Pneumonia, stomachache (Ssozi et al, 2016) |
|  |  |  | Bk | Syphilis | Boil, drink 1/2 cup 2 x daily |  |  | |  |
| *Tragia benthamii* Baker | Kamyu (AS157) | Herb | R | Sexual dysfunction | Decoction drunk | **W/A** | 3.5 | | Gonorrhea, malaria, ease child delivery |
|  |  |  | L | Vomiting, Diarrhoea | Decoction drunk | |  | | Reddy et al, 2017) |
|  |  |  | L | Toothache | Chew |  |  | |  |
|  |  |  | L | Eye infection | Pound, smear on eye lids | |  | |  |
|  |  |  | L | Athlete’s foot | Feet washed with infusion | |  | |  |
|  |  |  | L | Cervical wounds | Pound, add water, sit in extract | |  | |  |
| **Fabaceae**  *Abrus canescens* Baker. | Olusiiti (AS18) | Herb | Wp | Skin infections | Dry, pound, mix with jelly & apply on body | **W/R** | 3.5 | | Sickle cell (Amujoyegbe et al, 2016) |
| *Acacia hockii*  De Wild. | Akasana (AS032) | Tree | L | Wounds | Steam extract applied | **W/A** | 3.5 | | Athlete’s foot (Namukobe et al, 2011) |
|  |  |  | Fl, L | Ear infection | Pound, apply in ear | |  | |  |
| *Acacia polyacantha* Wild. | Kibeere (AS158) | Tree | Bk | Cough | Pound, dry, add water, boil + rock salt or ginger, drink | **W/A** | 3.5 | | antibacterial (Bhiwankar et al, 2015); diabetes (Okpanachi et al 2012) |
| *Albizia coriaria* Welw | Omugavu (AS005) | Tree | Bk | Back pain, Cancer | Boil for 3 hrs, drink for 9 days | **W/R** | 25 | | TB (Bunalema et al, 2013) |
|  |  |  | Bk | Skin infections | Decoction bathed & drunk | | | | bacterial & fungal infections (Asiimwe et al , 2013) |
|  |  |  | Bk | Athlete’s foot | Boil, soak feet |  |  | |  |
|  |  |  | Bk | Cough | Decoction drunk | |  | |  |
|  |  |  | Bk | Wounds (2) | Decoction bathed | |  | |  |
|  |  |  | Bk | Syphilis, Anaemia | 1/2 cup of decoction drunk twice daily | | | |  |
| *Cajanus cajan* L. Millsp. | Enkolimbo (AS080) | shrub | L | Gonorrhea | Decoction drunk | **C/A** | 3.5 | | sore eyes, measles (Tantengco et al, 2018) |
| *Crotalaria spinosa* Benth. | Kasambandege (AS159) | Herb | L | Paronychia | Pound, tie on affected area | **C/R** | 3.5 | | No literature |
| *Desmodium adscendens* (Sw.) DC. | Mutasuka kubo (AS160) | creeping herb | L | Cough | Decoction drunk | **W/A** | 7.1 | | No literature |
|  |  |  | L | Prevent miscarriage | Decoction drunk | |  | |  |
|  |  |  | L | Menorrhagia | Infusion bathed | |  | |  |
| *Entada abyssinica* A. Rich | Omuwolola (AS161) | shrub | Bk, L | Skin infections (5) | Pound, Boil, drink & bathe | **W/A** | 18 | | Edema (Kigen et al, 2017) |
|  |  |  | L | Wounds (5) | Decoction bathed | |  | | TB (Bunalema et al, 2013) |
|  |  |  | Bk | Syphilis | Decoction drunk & bathed | |  | | Gonorrhea (Yirga, 2010) |
| *Eriosema psoraleoides* (Lam) G. Don | Akakolimbo (AS162) | Herb | L | Skin infections | Decoction bathed | **W/A** | 3.5 | | No literature |
|  |  |  | L | Constipation | Decoction drunk | |  | |  |
| *Erythrina abyssinica* DC. | Ejirikiti (AS079) | Tree | Bk,L | Cough (9), Pressure | Boil, drink; chew with salt | **W/A** | 46.4 | | backache, wounds (Maroyi, 2011) |
|  |  |  | L | Fever & Malaria | Pound, add water, drink & bathe | | | |  |
|  |  |  | Bk | Skin infections (3) | Pound, Boil & bathe | |  | |  |
|  |  |  | Bk | Back pain, syphilis (4) | Decoction drunk | |  | |  |
|  |  |  | Bk | Blocked fallopian tubes | Decoction drunk | |  | |  |
|  |  |  | Bk,L | Diarrhoea (2) | Infusion/decoction drunk | |  | |  |
|  |  |  | Bk | Prevent miscarriage | Boil, drink and bathe | |  | |  |
| *Indigofera spicata* Forssk*.* | Mukaliza (AS163) | Herb | L | Syphilis | Decoction drunk | **W/A** | 3.5 | | diarrhoea (Woldeab et al 2018) |
| *Piptadeniastrum africanum* (Hook.f) Brenan | Empewere (AS164) | Tree | L | Eye infection (2) | Roast and squeeze, apply extract | **W/R** | 50 | | TB (Bunalema et al, 2013); cough (Ssegawa et al,2007) |
|  | |  | Bk | Meningitis , Cancer | Boil for 3 hrs, drink for 9 days | | | |  |
|  |  |  | Bk, L | Cough (5), TB | Boil with bark of *Syzigium cumini*  and *Mangifera indica*, drink | | | | |
|  |  |  | Bk | Painful bones | Decoction drunk | |  | |  |
|  |  |  | Bk | Heart palpitations | 250ml -500ml of decoction drunk daily | | | |  |
|  |  |  | Bk | Dizziness | 250ml -500ml of decoction drunk daily | | | |  |
|  |  |  | Bk | Malaria, Asthma | 250ml -500ml of decoction drunk daily | | | |  |
|  |  |  | L | Diarrhoea (4) | Infusion drunk |  |  | |  |
|  |  |  | L, Bk | Syphilis (2) | Boil, drink or bathe | |  | |  |
|  |  |  | L, Bk | nausea/  stomachache | Infusion drunk |  |  | |  |
|  |  |  | Bk | Boils, Anemia | Decoction drunk 3x for 9 days | |  | |  |
| *Phaseolus lunatus* L. | Akayindiyindi (AS165) | Herb | L | Cough (4) | Chew/ squeeze + ash+ salt, drink | **C/A** | 39.3 | | Spasm, stomachache |
|  |  |  | L | Eye infection (5) | Boil, drop in eyes | |  | | Tantengco et al, 2018) |
|  |  |  | L | Sore throat | Steam, squeeze and drink extract | | | |  |
|  |  |  | L | Sinus | Pound fresh with rock salt warm in clean cloth, drink | | | | |
|  |  |  | L | Tuberculosis, Nausea | Decoction drunk | |  | |  |
|  |  |  | Wp | Luck | Decoction bathed | |  | |  |
|  |  |  | L, Bk | Flue | Boil/squeeze, add ash + salt, drink | | | | |
| *Pseudarthria hookeri* Wight & Arn. | Ekikakala (AS058) | Herb | Bk | Stomach infections | Decoction drunk | **W/R** | 10.7 | | hernia (Kibuuka & Anywar, 2015) |
|  |  |  | L | Syphilis | Boil, drink & bathe | |  | |  |
|  |  |  | L | Fever | Pound, boil, drink | |  | |  |
| *Senna occidentalis* (L.) Link | Mutanjoka (AS166) | Shrub | R | Diarrhoea | Decoction drunk | **C/A** | 3.5 | | Stomachache, malaria |
|  |  |  | R | Deworming | Powder decoction drunk | |  | | cough (Appiah et al, 2018) |
| *Vigna unguiculata* L. Walp. | Ekiyindiru (AS167) | Herb | L | Syphilis | Decoction drunk & bathed | **W/R** | 3.5 | | antibacterial (Kritzinger & Aveling, 2004) |
|  |  |  |  |  |  |  |  | | epilepsy, jaundince (Sayeed et al, 2017) |
| **Hypericaceae** |  |  |  |  |  |  |  | |  |
| *Harungana madagascariensis* Lam. Ex. Poir | Omulirira (AS150) | Tree | Bk | Stomach infections | Decoction drunk | **W/R** | 3.5 | | Cancer (Soladoye, et al,2010) |
| *Psorospermum febrifugum* Spach | Akanzironziro (AS148) | Herb | L, Bk | Syphilis (3) | Burn, lick ash; mix powder with jelly & smear | **W/A** | 32.1 | | Skin rash (Nambejja et al, 2019) |
|  |  |  | Bk, R, L | Stomachache, Ulcers | Decoction drunk | |  | |  |
|  |  |  | Bk, R | Skin infections (6) | Powder mixed with jelly, smeared | | | |  |
|  |  |  | Bk | Athlete’s foot | Boil, soak feet |  |  | |  |
|  |  |  | Bk | Dysentery, malaria | Decoction drunk | |  | |  |
|  |  |  | L | Cough | Ash licked |  |  | |  |
| **Lamiaceae** *Hoslundia opposita* Vahl. | Kamumye (AS008) | shrub | L | Stomachache (4) | Macerate, drink infusion | **W/R** | 71.4 | | Stomachache, jaundice (Ssozi e al, 2016) |
|  |  |  | L | Nausea, Diabetes | Decoction drunk | |  | | Cataract (Maroyi 2011) |
|  |  |  | L | Wounds (4) | Macerate in hot water, squeeze, drink or apply topically | | | | postnatal care, wounds |
|  |  |  | L | Skin infections | Squeeze or dry, add vaseline, smear | | | | (Ogwal 1996; Okullo et al, 2014) |
|  |  |  | L | Jaundice | Mix with *Fleuggea virosa*, boil, drink | | | | Hepatoprotective, |
|  |  |  | L | Ulcers, Pressure (2) | Decoction drunk |  |  | | CNS depression (Said, 2018) |
|  |  |  | Bk | Syphilis (5) | Decoction drunk & bathed | |  | | Malaria (Appiah et al, 2018) |
|  |  |  | L | Memory boost, flue | Decoction drunk | |  | | Cough, TB (Asiimwe et al, 2013) |
|  |  |  | L | Gonorrhea (2), TB | Decoction drunk | |  | |  |
|  |  |  | Bk | Cough, back pain | Boil, drink 3 TS daily for 9 days | | | |  |
|  |  |  | L | Boils (2) | Steam in hot ash, squeeze and drink & apply on boil | | | | |
|  |  |  | L | Tumors | Heat on top of food, apply on tumor | | | | |
|  |  |  | L | Eye infection (6) | Roast and squeeze, apply extract in eyes | | | | |
|  |  |  | L | Sinus | Pound fresh with rock salt warm in clean cloth, inhale | | | | |
|  |  |  | Bk | Witchcraft | Decoction drunk | |  | |  |
|  |  |  | Bk | Arthritis | Boil for 3 hrs, drink for 9 days | | | |  |
|  |  |  | L | Postnatal care | Infusion drunk |  |  | |  |
| *Hyptis pectinata* (L.) Poit | Lukowe (AS168) | shrub | L | Fever | Squeeze in water, bathe | **W/A** | 3.5 | | Malaria, convulsions, (Ajibesin et al, 2012) |
| *Ocimum basilicum* L. | Akakubansiri (AS169) | Herb | L | Sore throat, Flue | Roast and squeeze, swallow extract | **W/R** | 7.1 | | Cold, malaria, antibacterial |
|  |  |  |  |  |  |  |  | | Diuretic (Sivasankari et al, 2014) |
| *Ocimum suave* Willd. | Omujaja (AS059) | Herb | L | Stomach infections /Antiemetic | Boil and drink hot | **C/A** | 3.5 | | Analgesic, antibiotic (Tan et al, 2005) |
|  |  |  | L | Diarrhoea | Slice and add to hot water, macerate and drink | | | | |
| *Plectranthus barbatus* Andrews | Ekibankulata (AS105) | Herb | Fl, L | Ear infection | Roast in banana leaves, drop in ear | **W/C/R** | 28.6 | | TB (Bunalema et al, 2013) |
|  |  |  | L | Cough, Malaria | Decoction drunk | |  | | Nausea (Hamil et al, 2003) |
|  |  |  | L | Tuberculosis | Infusion drunk |  |  | |  |
|  |  |  | Bk | Syphilis (2), Meningitis | Decoction drunk | |  | |  |
|  |  |  | L | Wounds (3) | Pound/roast & squeeze, apply extract | | | | |
|  |  |  | Bk | Cancer, arthritis, Back pain | Boil for 3 hrs, drink for 9 days | | | |  |
| *Tetradenia riparia* (Hochst.) Codd | Ekyewamala (AS075) | Herb | Bk | Cough (7) | Boil & drink; or dry, add salt & eat powder | **W/C/R** | 43 | | TB (Bunalema et al, 2013) |
|  |  |  | Bk | Painful bones | Decoction drunk | |  | |  |
|  |  |  | Bk | Heart palpitations | Boil, drink 1 cup daily | |  | |  |
|  |  |  | Bk | Dizziness | Boil, drink 1 cup daily | |  | |  |
|  |  |  | Lsap | Wounds | squeeze & apply | |  | |  |
|  |  |  | L | Eye infection (2) | Boil, add salt, apply in eyes | | | |  |
| **Lauraceae**  *Persea americana* Mill | Keddo (AS063) | Tree | L, Bk, Sd | Anaemia (5) | Dry & pound, Add powder to hot water; or boil, and drink | **C/A** | 32.1 | | Fever (Appiah et al 2018) |
|  |  |  | L | Cough (2) | Dry the leaves, chew powder with salt | | | | Diabetes |
|  |  |  | Sd | Paralysis, HBP | Dry, pound, add hot water & drink | | | | Diarrhoea (Woldeab et al, 2017) |
| **Loganiaceae** *Strychnos sp* | Ekobe (AS170) | Herb | R | Stomachache | Decoction drunk | **W/R** | 3.5 | | Antibacterial, antioxidant (Imam Isa et al 2014) |
| **Lythraceae**  *Punica granatum* L. | Nkomamawanga (AS171) | Tree | R | Diabetes | Decoction drunk | **C/A** | 3.5 | | Syphilis, cancer, bronchitis |
|  |  |  | Fr | Anaemia | Fruits eaten |  |  | | Nose bleed (Bhowmik et al, 2012) |
| **Malvaceae**  *Abutilon mauritianum* (Jacq.) Medik | Kifuula (AS 172) | Herb | L | Stomachache | Infusion drunk | **W/A** | 3.5 | | Ascariasis (Hamil et al, 2003) |
| *Hibiscus fuscus* Garcke | Olusaala (AS173) | shrub | L | Asthma | Dry, burn to ash, lick | **W/A** | 3.5 | | TB, cough, antiemetic |
|  |  |  |  |  |  |  |  | | Bunalema et al 2014; Ramathal & Ngassapa 2001) |
| *Hibiscus sabdariffa* L. | Ekyomusaayi (AS004) | Herb | Bk | Malaria, Anaemia | Boil, drink 1 cup daily | **C/R** | 3.5 | | HBP, cancer, obesity |
|  |  |  |  |  |  |  |  | | Helminthic ( Singh et al, 2017) |
| *Hibiscus surattensis* L. | Omuyeye (AS174) | Herb | Bk | Hernia | Decoction drunk | **W/R** | 3.5 | | Sores, malaria, STIs, cough |
|  |  |  |  |  |  |  |  | | wounds, HBP ( Raghu, 2015) |
| *Sida cordifolia* L. | Akeyeyo (AS175) | Herb | Bk, L | Asthma, Diabetes | Boil, drink 1 cup daily | **C/A** | 10.7 | | Cuts, wounds (Aravindran & Rajendran, 2013) |
|  |  |  | L | Wounds | Pound, apply extract | |  | |  |
|  |  |  | L | Headache | Drink 1 cup of infusion daily for a week | | | | |
|  |  |  | L | Convulsions | Squeeze in water while plant is still standing, bathe | | | | |
|  |  |  | L | Broken bones | Pound, dry, mix with oil, massage | | | |  |
| **Menispermaceae** *Chasmanthera dependens* (Hochst.) | Lubowa (AS176) | Herb | R, L | Malaria, Barrenness (2) | Decoction drunk | **C/A** | 7.1 | | Convulsions, epilepsy, dementia, |
|  |  |  |  |  |  |  |  | | Snake bites, malaria (Mosango, 2008) |
| *Cissampelos mucronata* A. Rich | Kavamagombe/kavawala (AS177) | creeping herb | R | Malaria | Decoction drunk | **W/A** | 14.3 | | Malaria (Ngarivhume et al, 2015; |
|  |  |  | L | Headache | Drink 1 cup of infusion daily | | | | Adia et al, 2014) |
|  |  |  | R | Cough (2) | Pound, dry, add water, boil + rock salt or ginger, drink | | | | |
| **Moraceae** *Artocarpus heterophyllus* Lam. | Fene (AS178) | Tree | Bk, L | Cough (3) | Boil, drink; dry the leaves, chew powder with salt | **C/A** | 7.1 | | Wounds (Tantengco et al, 20018) |
|  |  |  | L | Fever | Decoction drunk | |  | | Skin, laxative, ophthalmia |
|  |  |  | Sd | Ulcers | Dry, pound, drink with tea | |  | | (Sivasankari et al, 2014) |
| *Ficus natalensis* Hochst. | Omutuba (AS179) | Tree | L | Syphilis | Boil, drink 1/2 cup x 2 daily | **C/A** | 3.5 | | Bladder diseases (Ssozi et al, 2016) |
| *Ficus exasperata* Vahl | Oluwawu (AS180) | Shrub | L | Malaria | Infusion drunk | **W/C/A** | 3.5 | | malaria, shingles, wounds, Stomachaches (Appiah et al, 2019) |
| *Milicia excelsa* (Welw) C.C. Berg | Muvule (AS181) | Tree | Bk | Gonorrhea, Syphilis (2) | Decoction drunk | **W/C/R** | 18 | | Cough, wounds, heart (Ssozi et al, 2016) |
|  |  |  | L, Bk | Cough (2) | Dry the leaves, chew powder with salt | | | |  |
| **Moringaceae** *Moringa oleifera* Lam | Moringa (AS057) | Tree | L | Wounds, Sinus, Asthma | Dry or squeeze and drink | **C/A** | 3.5 | | Low blood pressure |
|  |  |  |  |  |  |  |  | | Cough (Tantengco et al, 2018) |
| **Musaceae**  *Musa paradisiaca* L. | Embide (AS182) | Tree | L | Cough , Vomiting | Decoction drunk | **C/A** | 3.5 | | Diarrhoea, toothache, (Tantengco et al, 2018) |
| **Myricaceae**  *Myrica kanditiana* Engl. | Kikimbo (AS183) | Herb | L | Syphilis | Decoction drunk | **W/A** | 3.5 | | TB (Bunalema et al, 2014) |
| **Myrtaceae** *Callistemon citrinus* (Curtis) Skeels. | Nyambala butonya (AS074) | Tree | L, Bk, Fl | Cough (4) | Pound, dry, add water, boil + rock salt or ginger & drink 3x daily | **C/A** | 14.3 | | TB (Bunalema et al, 2014) |
|  |  |  | L, Bk, Fl | Sinuses | Boil while covered, inhale steam | | | | Cough (Namukobe et al, 2011) |
| *Eucalyptus sp* | Kalitunsi (AS047) | Tree | Bk, L | Cough (2) | Pound, dry, add water, boil + rock salt or ginger, drink | **C/A** | 10.7 | | Malaria ( Adia et al, 2014) |
|  |  |  | R, Bk | Ulcers | Decoction drunk | |  | |  |
|  |  |  | Bk | Skin infections | Pound, mix with jelly, smear | | | |  |
| *Psidium guajava* L. | Amapeera (AS066) | Tree | L. Bk | Cough (6) | Boil, drink 3x daily; dry and chew powder with salt | **C/A** | 39.3 | | Cough, fever (Maroyi, 2011) |
|  |  |  | L | Fever | Decoction drunk | |  | | Malaria ( Adia et al, 2014) |
|  |  |  | L,R | Skin infections (4) | Pound, add jerry, smear | |  | |  |
|  |  |  | Bk | Athlete’s foot | Boil, soak feet |  |  | |  |
|  |  |  | L | Syphilis | Pound, dry, mix with oil & apply on body | | | | |
| *Syzygium cumini* (L.) Skeels | Jambula (AS184) | Tree | L, Bk | Cough (6) | Pound, dry, add water, boil + rock salt or ginger & drink | **C/R** | 36 | | Malaria ( Adia et al, 2014) |
|  |  |  | L | Sore throat | Chew |  |  | | Diabetes (Ssozi et al, 2016) |
|  |  |  | L | Dysentery | Decoction drunk | |  | |  |
|  |  |  | L | Syphilis, wounds | |  |  | |  |
|  |  |  | Wp | Skin infections, wounds | Dry, pound, mix powder with oil & smear | | | | |
| **Nyctaginaceae** *Boerhavia diffusa* L. | Katebekataka (AS185) | Herb | L | Blocked Fallopian tubes | Decoction drunk | **W/R** | 7.1 | | Diabetes, cancer  (Mahesh et al, 2012) |
|  |  |  | L | Boils | Steam extract applied | | | |  |
| ***Phyllanthaceae*** |  |  |  |  |  |  |  | |  |
| *Flueggea virosa* (Roxb.Ex. Willd) Royle | Olukandwa (AS028) | Shrub | L | Skin infections | Squeeze or dry, add vaseline, smear | **W/A** | 3.5 | | Pneumonia, contraceptive (Maroyi, 2011) |
| *Phyllanthus capillaris* Schumach & Thonn | Omutulika (AS155) | Herb | L | Skin infection | Decoction drunk | **W/A** | 21.4 | | measles (Namukobe et al, 2011) |
|  |  |  |  | Measles (5) | Boil with small fish | |  | |  |
| *Phyllanthus amarus*  Schum. & Thonn | Nakitembe (AS156) | Herb | L | Stomachache/  Antenatal care | Infusion drunk | **W/A** | 3.5 | | TB (Bunalema et al ,2014) |
| **Phytolacaceae** *Phytolacca dodecandra* L Her | Luwoko (187) | shrub | R | Gonorrhea | Infusion drunk | **W/A** | 3.5 | | antimalarial (Karunamoorti & Tsehaye, 2012) |
| **Poaceae** *Pennisetum purpureum* Schumach. | Ekisagazi (AS188) | shrub | L | Gonorrhea | Infusion drunk | **W/A** | 7.1 | | Nutrition (Okoraonye & Ikewuchi 2009) |
|  |  |  | L | Headache | Drink 1 cup daily of infusion for a week | | | | |
| **Polygonaceae** *Oxygonum sinuatum* (Hochst & Steud ex Meisn) Dammer | Kafumita bagenge (AS189) | Creeping herb | L | Boils (2) | Steam extract applied | **W/A** | 21.4 | | Bacterial skin infections, gonorrhea |
|  |  |  | L | Tumors | Steam applied on tumor | | | | (Ramathal & Ngassapa, 2001)) |
|  |  |  | L | Convulsions | Squeeze in water while plant is still standing, bathe | | | | |
|  |  |  | L | Diarrhoea | Infusion drunk |  |  | |  |
|  |  |  | Fr | Ear infection | Roast, apply extract in ear | | | |  |
| **Portulacaceae** *Portulaca grandiflora* Hook. | Bwanda (AS190) | Herb | L | Wounds, Stomachache | Macerate in hot water, drink | **W/R** | 7.1 | | Sore throat, skin rash, hepatitis |
|  |  |  |  |  |  |  |  | | Burns (Adriana et al, 2013) |
| **Ranunculaceae** *Clematis sinensis* Lour | Lumaama (AS191) | Climbing herb | L | Skin infections | Decoction bathed | **W/A** | 3.5 | | wounds,  Stomachache (Admasu & Yohannes, 2019) |
| **Rhamnaceae** *Maesopsis eminii* Engl. | Omusizi (AS192) | Tree | Bk | Diarrhoea | Decoction drunk | **C/A** | 3.5 | | Diabetes (Situmorang et al, 2015) |
| **Rosaceae** *Eriobotrya japonica* (Thunb.) Lindl | Ensali (AS193) | Tree | Bk | Syphilis | Boil, bathe & drink 1/2 cup | **W/R** | 3.5 | | cough, cancer, Diabetes, asthma (Singh et al, 2010) |
| *Rubus rigidus* Sm | Enkenene (AS194) | shrub | Bk | Stomachache | Boil, drink 1/2 cup | **W/R** | 7.1 | | hernia (Kibuuka & Anywar, 2015) |
|  |  |  | L | Sore throat | Chew |  |  | |  |
| **Rubiaceae**  *Coffea canephora* | Emwanyi (AS195) | shrub | L | Sore throat | Chew | **C/A** | 7.1 | | Jaundice, HBP, deworming |
| Pierr ex A. Froehner | |  | Bk | Cough | Pound, dry, add water, boil + rock salt or ginger, drink | | | | Carrios & Valles 2012) |
| *Rubia cordifolia* L. | Kasalabakesi (AS196) | Herb | L, Wp | Cough (2) | Dry, burn, add salt, lick ash | **W/A** | 3.5 | | TB (Bunalema, 2014) rheumatism (Gireesha & Raju, (2013) |
| *Vangueria apicultata* K. Schum. | Matugunda (AS197) | Tree | L | Nausea | Decoction drunk | **C/A** | 7.1 | | GIT, toothache, antidote, Hiccups (Okullo et al, 2014) |
| **Rutaceae**  *Citrus limon*  (L) Osbeck | Enimu (AS198) | Shrub | Fr | Cough | Eat or boil the peels with honey, drink | **C/A** | 3.5 | | HBP, laxative, headache, deworming, (Carrios & Valles, 2012) |
| *Toddalia asiatica* (L) Lam. | Kawule (AS199) | shrub | Bk | Anti - poison | Pound, add water, drink | **C/W/A** | 3.5 | | TB (Bunalema et al, 2014) |
|  |  |  | Bk | Stomach infections | Pound, add water, drink | |  | |  |
|  |  |  | Bk | Cough | Pound, dry, add water, boil + rock salt or ginger, drink | | | | |
| *Zanthoxylum chalybeum* Engl. | Ntaleyaddungu (AS200) | Tree | Bk | Syphilis (2), Witchcraft | Decoction drunk | **W/R** | 14.3 | | TB (Tabuti et al, 2010) |
|  |  |  | Bk | Back pain (2), Cancer | Boil for 3 hrs, drink for 9 days | | | |  |
|  |  |  | Bk | Blocked fallopian tubes | Decoction drunk | |  | |  |
|  |  |  | L, R | Toothache | dry, use powder & tooth paste to brush teeth after dinner | | | | |
| *Zanthoxylum leprieurii* Guill & Perr. | Omunyenye (AS201) | Tree | Bk | Ulcers | Decoction drunk | **W/R** | 14.3 | | TB (Bunalema et al, 2017) |
|  |  |  | Bk | Cough, Gonorrhea | Decoction drunk | |  | | HIV/AIDS, malaria, rheumatic pain (Halim 1997) |
|  |  |  | Bk, L | Syphilis | Dry, boil powder, drink, bathe | | | |
| **Sapindaceae** *Cardiospermum halicacabum* L. | Olunyereketo (AS202) | Herb | L | Ulcers | Add water to powder, drink | **W/A** | 14.3 | | Nervous system, fractures |
|  |  |  | Wp | Skin infections (2) | Powder mixed with jelly applied on body | | | | Rheumatism, hemorrhoids |
|  |  |  | L | Stomachache | Infusion drunk | |  | | (Sivasankari et al, 2014) |
|  |  |  | L | Wounds, Measles | Extract applied | |  | |  |
| **Solanaceae** *Nicotiana tabacum* L. | Tabba (AS203) | Herb | L | Cough | chew | **C/R** | 3.5 | | Wounds (Maroyi 2011) |
| *Physalis angulata* L. | Akatuntunu akatono (AS204) | Herb | Fl, L | Ear infection | Roast in banana leaves, drop in ear | **W/A** | 7.1 | | Hepatitis, toothache, rheumatic pain (Mairura, 2008) |
|  |  |  | Bk | Cough | Decoction drunk | |  | |  |
| *Physalis peruviana* L. | Entuntunu (AS077) | Herb | L, Bk | Cough (3) | Decoction drunk; powder chewed with salt | **C/A** | 7.1 | | Malaria (Adia et al, 2014) |
| *Solanum campylacanthum* Hochst. | Akatengotengo akatono (AS205) | Herb | Fr | Ear infection (2) | Drops of steam applied in ear | **W/R** | 7.1 | | Pre-hepatic jaundice (Ssegawa et al 2007) |
|  |  |  | R, Bk | Diarrhoea, Cough | Decoction drunk | |  | |  |
| *Solanum dasyphyllum* Schumach & Thonn. | Olutengotengo (AS206) | Herb | R | Witchcraft | Steam and chew | **W/A** | 3.5 | | Cough, trypanasomiasis (Bekalo et al, 2009) |
| *Solanum lycopersicum* L. | Akanyanya akatono (AS207) | Herb | Bk , L | Cough, Diarrhoea | Infusion drunk | **C/R** | 7.1 | | Facial spots (Abbasi et al, 2010); measles, convulsions, Appiah et al, 2019) |
| **Urticaceae**  *Fleurya aestuans* (L.) Gaudich | Omunyango (AS208) | Herb | L | Fibroids | Decoction drunk | **W/R** | 3.5 | | TB (Bunalema et al, 2013) |
| **Verbenaceae** *Lantana trifolia* L. | Akayukiyuki (AS049) | shrub | L | Eye infection | Salty extract  Dropped in eyes | **W/A** | 10.7 | | Rheumatic, malaria |
|  |  |  | L | Cough, Flue | Decoction drunk |  |  | | Tetanus (Sivasankari et al, 2014) |
| *Priva adhaerens* (Forssk.) Chiov. | Enkami (AS209) | Herb | L, Bk | Syphilis (3) | Decoction drunk & bathed | **W/R** | 28.6 | | Diarrhoea (Nansunga et al, 2014; Anokbongo et al, 1990) |
|  |  |  | Bk | Cancer, Inflammation, Boils, Anemia, Stomachache Convulsions, | Decoction drunk | |  | |  |
| **Vitaceae** *Cyphostemma adenocaule* | Akabombo akatono (AS113) | creeping herb | L, Wp | Syphilis (2) | Infusion drunk | **W/C/R** | 21.4 | | Vegetable, ophthalmia, sore throat, |
| (Steud.ex. A. Rich)  Desc.ex. Wild & R. B. Drumm | | | Wp | Wounds | Decoction drunk | |  | | Malaria (Bosch, 2004) |
|  |  |  | R | Skin infections (3) | Boil, bathe or smear with jelly | | | |  |
|  |  |  | L | Paronychia | Steam applied on affected area | | | |  |
|  |  |  | L | Chest pain | Pound, filter, warm, sit in extract | | | |  |
|  |  |  | Bk | Sexual dysfunction, Stomachache | Boil, drink 1/2 cup | |  | |  |
| *Cyphostemma ukerewense* (Gilg.) Desc. | Kikonyogo (AS210) | Herb | L | Witchcraft | Decoction drunk | **C/A** | 3.5 | | Tonsils (Odongo et al, 2018) |
| **Xanthorrhoeaceae**  *Aloe Vera (*L.) Burm.f | Akagaji akatono (AS107) | Herb | L | Cough (2) | Decoction / Infusion drunk | **C/A** | 36 | | HIV/AIDS care (Kisangau et al, 2011) |
|  |  |  | L, R | Malaria (4) Fever (2) | Decoction / Infusion runk | |  | |  |
|  |  |  | L | Meningitis, Brucella | Infusion drunk |  |  | |  |
|  |  |  |  | Skin infections | Decoction bathed | |  | |  |
| Column 1: Plant family & Scientific names; Colum 2: Local name (Luganda), plant collection number; Column 4: Parts used - L, Leaves; Bk- stem bark; Fr- fruits, Sd- seeds; Fl- flowers; R-roots; wp-whole plant; Column 5: Ailments (use reports per informant); Column 7: Conservation status - W-wild; C- Cultivated; R-rare; A-Abundant; | | | | | | | | | |
| Column 8: Percent respondent knowledge; Column 9: Literature cited for the plants in other previous studies | | | | | | | | | |
